# Development and Characterization of Chitosan and Chondroitin Sulfate Based Hydrogels Enriched with Garlic Extract for Potential Wound Healing/Skin Regeneration Applications

**DOI:** 10.3390/gels8100676

**Published:** 2022-10-20

**Authors:** Fatima Masood, Muhammad Atif Makhdoom, Iftikhar Ahmed Channa, Sadaf Jamal Gilani, Ahmad Khan, Rabia Hussain, Syeda Ammara Batool, Kiran Konain, Saeed Ur Rahman, Abdul Wadood, May Nasser bin Jumah, Muhammad Atiq Ur Rehman

**Affiliations:** 1 Department of Materials Science and Engineering, Institute of Space Technology, Islamabad 44000, Pakistan; 2 Institute of Metallurgy and Materials Engineering, University of the Punjab, Lahore 54590, Pakistan; 3 Thin Film Laboratory, Department of Metallurgical Engineering, NED University of Engineering and Technology, Off University Road, Karachi 75270, Pakistan; 4 Department of Basic Health Sciences, Preparatory Year, Princess Nourah Bint Abdulrahman University, Riyadh 11671, Saudi Arabia; 5 Molecular Biology, Institute of Basic Medical Sciences, Khyber Medical University, Peshawar 25120, Pakistan; 6 Oral Biology, Institute of Basic Medical Sciences, Khyber Medical University, Peshawar 25120, Pakistan; 7 Biology Department, College of Science, Princess Nourah Bint Abdulrahman University, Riyadh 11671, Saudi Arabia; 8 Environment and Biomaterial Unit, Health Sciences Research Center, Princess Nourah Bint Abdulrahman University, Riyadh 11671, Saudi Arabia; 9 Saudi Society for Applied Science, Princess Nourah Bint Abdulrahman University, Riyadh 11671, Saudi Arabia

**Keywords:** hydrogels, polymers, regeneration, antibacterial, drug release, garlic, alicin

## Abstract

Hydrogels can provide instant relief to pain and facilitate the fast recovery of wounds. Currently, the incorporation of medicinal herbs/plants in polymer matrix is being investigated due to their anti-bacterial and wound healing properties. Herein, we investigated the novel combination of chitosan (CS) and chondroitin sulfate (CHI) to synthesize hydrogels through freeze gelation process and enriched it with garlic (Gar) by soaking the hydrogels in garlic juice for faster wound healing and resistance to microbial growth at the wound surface. The synthesized hydrogels were characterized via Fourier-transform infrared spectroscopy (FTIR), which confirmed the presence of relevant functional groups. The scanning electron microscopy (SEM) images exhibited the porous structure of the hydrogels, which is useful for the sustained release of Gar from the hydrogels. The synthesized hydrogels showed significant inhibition zones against *Escherichia coli (E. coli)* and *Staphylococcus aureus (S. aureus)*. Furthermore, cell culture studies confirmed the cyto-compatibility of the synthesized hydrogels. Thus, the novel hydrogels presented in this study can offer an antibacterial effect during wound healing and promote tissue regeneration.

## 1. Introduction

Skin damage can occur in daily life due to the number of reasons. Small first-degree wounds heal rapidly, but second or third-degree wounds take time to recover and during the healing process the patient may face dehydration, infection, etc. The majority of third-degree wound patients are concerned with delayed healing issues and the scarring that results from the healing process [1]. Hydrogels are three-dimensional (3D) polymeric networks with a macro porous structure which allows for the favorable cellular interaction with the host tissue. The porosity in the hydrogels allows the diffusion and transportation of external nutrients and can also promote cellular metabolic processes [2,3]. Tissue engineering, drug delivery, and skin regeneration are just a few of the therapeutic applications of hydrogels [4,5,6]. Hydrogels can be gelled using either physical or chemical cross-linking processes [7]. Weak connections (covalent bonding) between polymer network allow the physical gelation [8,9,10]. The hydrogen bonding between the polymer chains achieved via a chemical cross-linking agent generates strong chemical bonds [11,12]. Chemically cross-linked hydrogels can resist permanent deformation, however, these types of hydrogels degrade under pressure [13]. Hydrogels can retain high water content and possess the delicate softness allowing for the hydrogels to mimic natural tissue [14]. Epichlorohydrin, glutaraldehyde, adipic acid dihydrazide and many other chemicals were used as a cross linker in [15]. In this study, glycerol is used as a cross linker.

Chondroitin sulfate (CHI) is a structural component found in connective tissues and cartilage [14]. CHI is made up of repeating D-glucuronic acid and N-acetyl galactosamine disaccharide units which are sulfated at 4- or 6-positions [16]. It is related to the glycosaminoglycans (GAGs), which are found mostly on the surface of the cells and in the extracellular matrix [17]. GAGs have a high viscosity and low compressibility, which makes them ideal material for joint lubrication [18]. Usually, a cross linker is used to adjust the characteristics of CHI [19] or it is mixed with other polymers to attain more stability, such as chitosan (CS) [14,20], gelatin, hyaluronic acid [21], collagen [22], poly(vinyl alcohol) [22] or poly- lactic-co-glycolic acid [23].

CS is a polysaccharide that abundantly comes from marine sources [24]. CS is also known as b-(1, 4)-2-acetamido-2-deoxy-D-glucose. CS is used in numerous biomedical applications because of its superior biocompatibility, biodegradability, non-toxicity, angiogenicity, and anti-bacterial capabilities at low molecular weight [25,26]. CS-based wound dressings have a number of unique features that make them effective for wound healing, including hemostasis [27], and antibacterial characteristics [28].

Allium sativum (garlic) is a natural plant that is widely used owe to bioactive compounds and remarkable wound healing properties. Allicin is the active component in a garlic aqueous extract [29]. Since ancient times, people have utilized garlic, one of the earliest known medicinal plants, to treat a number of human illnesses. Garlic can provide anti-inflammatory, antioxidant, antifungal, and wound healing properties owing to its favorable biochemical reactions [30].

Here, we report the CS/CHI/Gar-based hydrogels for the first time (to the best of our knowledge). The synthesized hydrogels can provide an antibacterial effect owing to allicin (a component of Gar) and can improved tissue regeneration/remodeling due to the structural similarities between CS/CHI with the extracellular matrix (ECM) of the soft tissues. The synthesized hydrogels presented favorable results in both cases. Thus, the synthesized hydrogels can be used to treat second- and third-degree wounds.

## 2. Results and Discussion

### 2.1. Morphological Analysis

**Figure 1:**
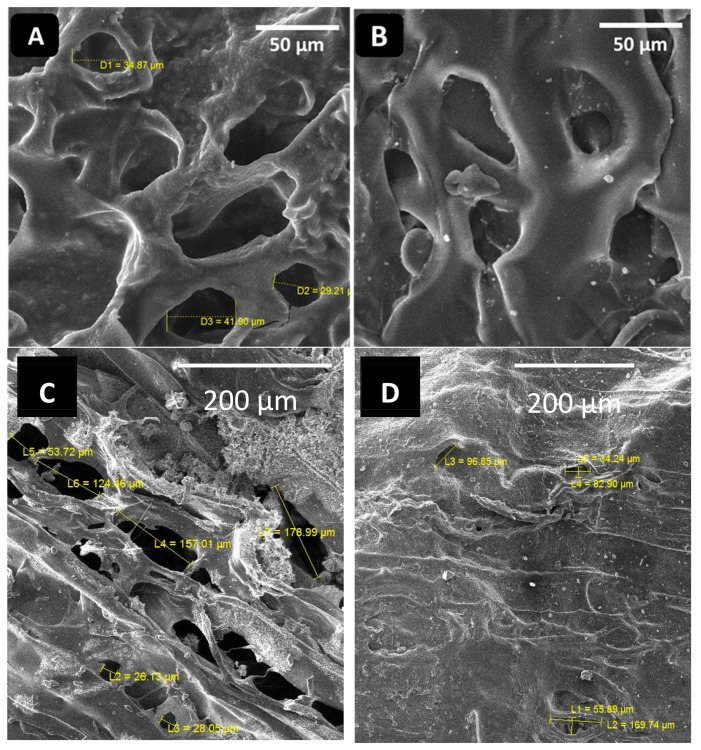
SEM images of (**A**) 80CS20CHI hydrogel and (**B**) 80CS20CHIGar hydrogel (**C**) 67CS33CHI hydrogel and (**D**) 67CS33CHIGar hydrogel.

The hydrogels were synthesized via cross-linking of NH_2_ and CHO groups of CS and CHI, respectively (while mixing in the presence of glycerol). SEM was used to confirm the morphology of the hydrogels. The porous structure of hydrogels is required in order to load the garlic molecules in the hydrogels effectively. The more porosity in the hydrogel means that more garlic can be loaded in it. Figure 1A,B shows the SEM images of the 80CS20CHI hydrogel before and after loading Gar, respectively. Figure 1C,D shows the SEM images of the 67CS33CHI hydrogel and 67CS33CHIGar hydrogel.

Porosity is a necessary aspect for hydrogels [31] and the SEM clearly indicate the porous nature of the synthesized hydrogels. These pores are vital as they help in retaining the water for long time and also help in the drug release. Additionally, the porosity of the hydrogels allows the exchange of nutrients and the skin regenerative factors to reach the wounded site [32]. The porous hydrogels were significantly more active than non-porous hydrogels and for the active skin regeneration model the average pore size should be between 20–125 µm [33]. The SEM results of this study are in agreement with where it was shown that the collagen-based hydrogels were more morphologically active at the 20–125 µm range, which is the favorable porosity and microstructure of the hydrogel [33]. The absorption of Gar in the hydrogel’s 3D-network was confirmed by swelling ratio by measuring the weight of hydrogel before and after loading Gar.

### 2.2. FTIR Analysis

**Figure 2:**
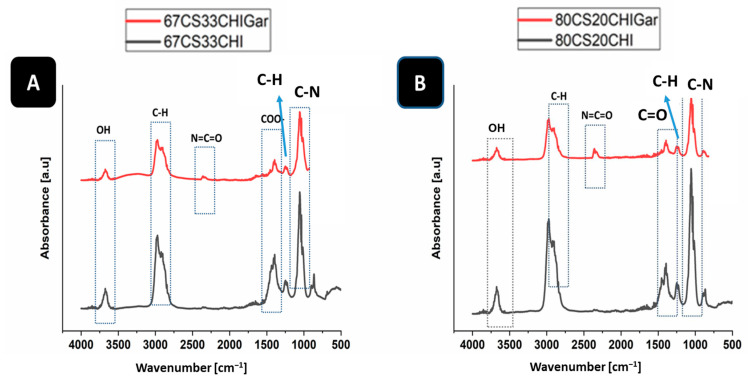
FTIR spectra of (**A**) 67CS33CHI, 67CS33CHIGar hydrogels and (**B**) 80CS20CHI, 80CS20CHIGar hydrogels.

FTIR was used to confirm the cross-linking of the hydrogel and Gars’ active components presence in the hydrogel by identifying the characteristic functional groups. Figure 2 shows the FTIR spectra of 67CS33CHI and 80CS20CHI hydrogels before and after loading Gar.

In the case of 67CS33CHI and 67CS33CHIGar hydrogels (Figure 2A), the absorption bands of CS at 2900 cm^−1^ [34] and 1062 cm^−1^ represent the C-H stretching vibrations and C-O stretching, respectively [35]. The absorption bands of CHI at 3670 cm^−1^ [36] and 1398 cm^−1^ [37] represent the stretching of OH group and COO- stretching of amino side chains, respectively. The absorption peaks of Gar at 2300 cm^−1^ [29] and 887 cm^−1^ [29] represent the isocyanate group of vibration (N=C=O) and C-H bending (mainly glycogen), respectively. Figure 2B shows the FTIR spectra of 80CS20CHI and 80CS20CHIGar hydrogels. The absorption bands of CS at 2900 cm^−1^ [38] and 1372 cm^−1^ [39] represent the C-H symmetric stretching and presence of hydroxyl group (OH), respectively, while the absorption bands of CS at 1015 cm^−1^ [37] represent the stretching of C-N bond. The absorption peaks of Gar at 2300 cm^−1^ [29] and 661 cm^−1^ [29] represent the isocyanate group of vibration (N=C=O) and C-Br stretching of alicyclic axial (bromo compounds), respectively.

### 2.3. Brunauer-Emmett-Teller (BET) Surface Area Analysis

**Figure 3:**
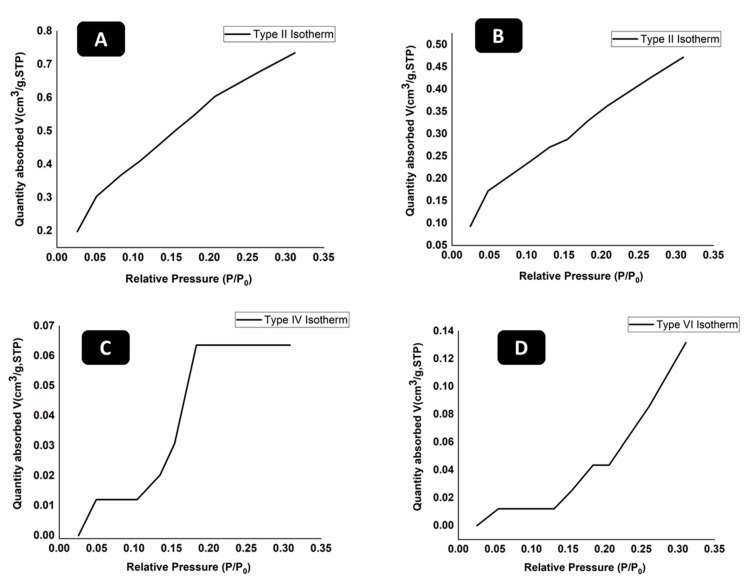
BET analysis of (**A**) 67CS33CHI, (**B**) 80CS20CHI, (**C**) 67CS33CHIGar, and (**D**) 80CS20CHIGar hydrogels.

BET was used to find the surface area and size of pores present in hydrogels. Different types of isotherms are obtained in BET and each has its own representation and explanation. BET was carried out for Gar loaded and unloaded samples. Figure 3 shows the BET results of all hydrogels.

Figure 3 represents the different types of adsorption isotherms obtained for different concentration of hydrogels before and after loading Gar. The typical isotherm formed with a non-porous or macro porous adsorbent is the reversible Type II isotherm (Figure 3A,B). Monolayer-multilayer adsorption without restriction is represented by the Type II isotherm [40]. In contrast to Type II isotherm, Type IV isotherm (Figure 3C) has a finite multi-layer formation that corresponds to the capillaries being completely filled. Adsorption comes to an end close to a relative pressure of unity. By adsorbing water vapor on activated carbon at 30 °C, this form of isotherm is created. This isotherm represents the macro-pore porosity in the hydrogel. The type VI isotherm (Figure 3D), also known as stepwise multilayer adsorption, only manifests when the sample surface has a variety of energetically diverse adsorption sites. Step formation in the adsorption isotherm represents layer by layer adsorption of nitrogen gas molecules [41].

The surface area of 67CS33CHI hydrogel was 2.53 m^2^/g and surface area of 80CS20CHI hydrogel was 1.65 m^2^/g. The surface area of 67CS33CHI hydrogel after 148 loading Gar decreased to 2.07 m^2^/g and for 80CS20CHIGar, area was 1.25 m^2^/g. It was noted that surface area decreased for both; 67CS33CHI and 80CS20CHI hydrogels after loading Gar which is an indication of presence of Gar juice inside the pores. In conclusion, the hydrogels with the garlic extracts were less porous than the hydrogels without garlic extracts. 

### 2.4. Swelling Behavior of Hydrogels

Swelling behavior of the hydrogels is vital for drug release and exchange of nutrients in tissue regeneration. To compare the water uptake capacity of the polymeric matrices, their swelling behavior was investigated. The swelling of the hydrogel is an essential component for determining its capacity for retaining water. Water-retaining hydrogels maintain the moisture at the wound site and can promote the healing process [42]. Hydrogels are considered most effective if they can retain water more than their initial weight. The maximum swelling ratio of hydrogels found up until now is 130 [43]. The hydrogels synthesized in this work absorbed water more than their initial weight. CS and CHI both contain hydrophilic groups in their polymer chains including hydroxyl, amino, and carboxyl groups which facilitated the abundant hydration of hydrogels [44]. Table 1 depicts the swelling percentages of both types of hydrogels. 67CS33CHI reached the swelling of ~2641%. It is inferred that 80CS20CHI hydrogel has a slightly higher swelling percentage, i.e., approximately up to 2675%. However, the difference is too small to make any impact. Statistical analysis of swelling percentages of synthesized hydrogels was carried out. It was observed that 67CS33CHI hydrogel and 80CS20CHI hydrogel are insignificantly different (at *p <* 0.05) in terms of their swelling percentages. 

### 2.5. In-Vitro Degradation Studies

The degradation behavior of hydrogels was monitored in terms of their % weight loss upon immersion in PBS. Hydrogels (synthesized in this study) were immersed in PBS solution for 21 days at 37 °C and the weight change of the hydrogels was measured to see the influence of hydrogel degradation [45]. It was observed that the weight of 67CS33CHI and 80CS20CHI hydrogels with and without Gar changed after 21 days, which indicated the degradation of hydrogels in PBS. Table 2 shows the difference in the weight before and after immersing in the PBS solution.

The highest weight loss was observed in the case of 67CS33CHIGar hydrogel reaching up to 91% due to the dissolution of hydrogel components in PBS with time. After this, 73% weight loss was measured for 67CS33CHI followed by 57% for 80CS20CHI and 18% for 80CS20CHIGar. The degradation difference between hydrogels indicates the difference of degree of cross-linking in hydrogels. Hydrogels with low cross-linking density under go faster degradation [46], as depicted by 67CS33CHIGar. The relatively higher degradation rate of hydrogels in this study is attributed to the presence of CHI [16]. The statistical analysis shows the significant difference of degradation between the two hydrogels at *p <* 0.05.

### 2.6. Antibacterial Analysis of Hydrogels

**Figure 4:**
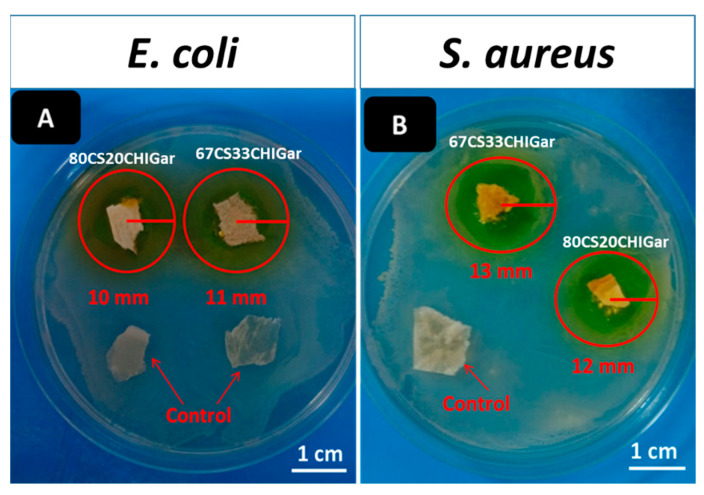
Inhibition zone around both hydrogels (**A**) *E. coli* and (**B**) *S. aureus*.

Antibacterial analysis of hydrogels was carried. Figure 4 shows the antibacterial results of hydrogels at different concentrations against *E. coli* and *S. aureus*. Figure 4A represents the two control hydrogel samples i.e., 67CS33CHI and 80CS20CHI hydrogels around which no inhibition zone was observed. However, 67CS33CHIGar and 80CS20CHIGar hydrogels show the inhibition zone of 11 mm and 10 mm, respectively. Figure 4B represents the 67CS33CHI hydrogel (control), which did not show any inhibition zone while 67CS33CHIGar and 80CS20CHIGar hydrogels show the inhibition zone of 13 mm and 12 mm, respectively. Thus, it was concluded that both concentrations work almost same against both bacterial strains. In comparison, 67CS33CHIGar hydrogel worked best among the two hydrogels with a slightly bigger zone of inhibition. Degradation study showed that degradation rate of 67CS33CHIGar is higher than all other hydrogels which means that more Gar components were released from 67CS33CHIGar which were more effective against *E. coli* and *S. aureus*.

### 2.7. Cell Culture Studies

**Figure 5:**
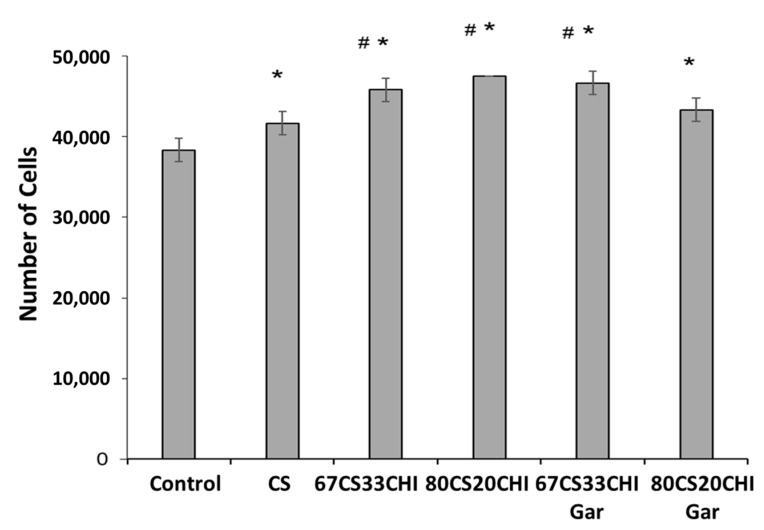
Response of fibroblast cells towards the different compositions of the hydrogels- * symbolizes the significant difference between control and other groups as *p* value is ≤0.05, # symbolizes the significant difference (*p* ≤ 0.05) between the CS and 67CS33CHI, 80CS20CHI and 67CS33CHI-Gar.

It is well known that the porosity of hydrogel can enhance the cells attachment and function [47]. The synthesized hydrogels were sufficiently porous in nature; hence, cell-studies were carried out on fibroblasts cell line to observe their interaction with hydrogels. The cells were cultured on hydrogels before and after loading Gar. Our results demonstrated that the cell attachment significantly increased on all hydrogels as compared to control, as depicted in Figure 5. The addition of CHI in the CS also had a positive influence on cell-adhesion and proliferation [48]. Moreover, our results showed that 67CS33CHI, 80CS20CHI, and 67CS33CHIGar hydrogels significantly increased cells adhesion as compared to CS alone. Among all hydrogels, the maximum number of cells were attached to 67CS33CHIGar which might be due to the highest surface area availability as discussed in the BET surface area calculations. Overall, our results suggested that hydrogels containing 67CS33CHIGar might be an ideal candidate for the induction of tissue regeneration. Taking together the results of the antibacterial and cell biology studies, it was concluded that the 67CS33CHIGar is the best composition for designing wound healing patches.

## 3. Conclusions

In this research, the garlic-loaded CS and CHI hydrogels were synthesized and examined for biological applications. Two different compositions, i.e., 67CS33CHI and 80C20CHI were synthesized and their properties were compared before and after garlic loading. Both compositions showed high swelling ratio and suitable degradation rate for drug loading and targeted release purposes. However, the 67CS33CHIGar composition was determined to have the greatest potential for biomedical applications, as it demonstrated excellent compatibility to fibroblasts cell line, cell-adhesion and proliferation, higher degradability, as well as better antibacterial activity against *E. coli* and *S. aureus* as compared to the 80C20CHIGar hydrogel.

## 4. Experimental Procedure

### 4.1. Materials

CS (M.W = 236934.2 g/moL, degree of de-acetylation [DD]: 83%, intrinsic viscosity 30.78 mL/g) and sodium salt of CHI extracted from shark cartilage was purchased from Sigma Aldrich^®^ (M.W = 475.4 g/mol). Analytical reagent grade sodium hydroxide was purchased from Omicron Sciences limited^®^. Acetic Acid (Glacial 100% extra pure), and ethanol (M.W = 46.07 g/moL) were purchased from Sigma Aldrich^®^. Glycerol (M.W = 92.10 g/moL) was purchased from Merck^®^. The garlic was purchased from the Imtiaz super store in Islamabad. All the chemicals were used as received without any further treatment or purification.

### 4.2. Synthesis of Hydrogels

**Figure 6:**
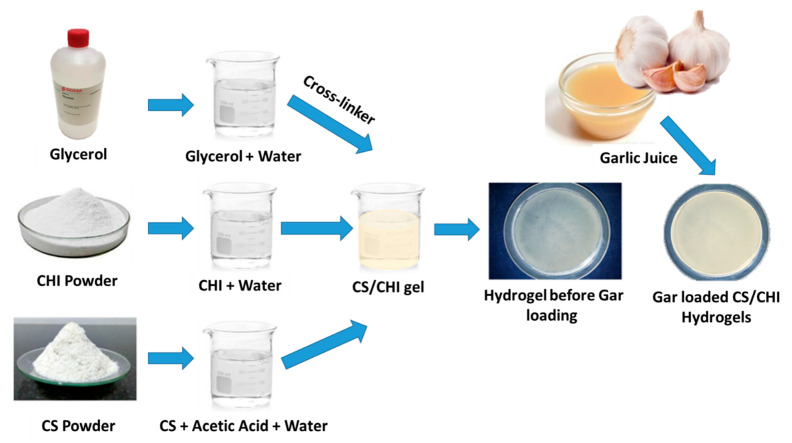
Synthesis mechanism of Gar loaded CS and CHI based hydrogels.

The CS solution was prepared by adding 1.15 mL of acetic into 98.95 mL of distilled water to make 100 mL solution. The solution was magnetically stirred for 5–10 min then 2 g of CS was added. In parallel, CHI solution was prepared by adding 2 g of CHI in 50 mL of distilled water and stirring until the homogeneous solution was formed. Glycerol was used as a cross linker. An amount of 1.5 mL glycerol was added in 98.5 mL of distilled water and was magnetically stirred for 5 min. In the next step, CS and CHI solutions (as shown in Table 3) were mixed in different ratios and stirred to get homogeneous solution followed by the addition of glycerol solution. Then, the two solutions were poured in separate Petri dishes and were kept at −20 °C for 4 days. After that, 3 molar NaOH solutions (6 g of NaOH in 50 mL ethanol) was poured on to the gels homogeneously and again kept in refrigerator at −20 °C for 48 h. Gel-washing was performed 3 times with ethanol solution (50 mL ethanol, 50 mL water) and the pH of the gels was measured until the pH of the gels was neutral. The weight of each gel was measured using a physical balance (Shimadzu-AUY220 accurate up to 10 mg). Gels were kept at room temperature for drying for almost 30–36 h and were then weighed again after drying. In the final step, Gar juice was extracted from raw garlic with the help of juicer. Prior to chopping, garlic cloves were washed with distilled water and air dried. The juice was obtained by grinding the garlic cloves along with the distilled water. To sterilize the mixture, it was kept under UV-light for 45 min. Gels were soaked in the Gar juice for 5–10 min and were dried at room temperature. Figure 6 shows the synthesis mechanism of the Gar loaded CS and CHI hydrogels.

Hydrogels with different compositions of CS/CHI/Gar were synthesized, however, the above-mentioned compositions worked best against various tests. The 50CS50CHI composition was also used, but it did not form the gel.

### 4.3. Materials Characterization

#### 4.3.1. Scanning Electron Microscopy (SEM)

The surface morphology of prepared hydrogels was investigated using the SEM (FE-SEM, MIRA, TESCAN). The samples were first coated with a thin layer of gold using a sputtering coater to make them conductive and diminish the effect of charging. SEM images were taken at low and high magnifications. Energy dispersive spectroscopy (EDS) was also performed to analyze the elemental composition of the hydrogels.

#### 4.3.2. Fourier Transformation Infrared Spectroscopy (FTIR)

The presence of different functional groups in synthesized hydrogels was investigated by FTIR (thermo-scientific Nicolet Summit LITE). FTIR measurements were carried out on both; the initial materials (CS/CHI/Gar juice) and the synthesized hydrogels (before and after loading garlic). After preparing the samples, the IR absorbance spectra were recorded in the range of 400–4000 cm^−1^.

#### 4.3.3. Brunauer-Emmett-Teller (BET) Surface Area Analysis (Textural Properties)

The surface area of prepared hydrogels was examined using the BET (V-Sorb 2800P, Beijing, China) surface area and porosimetry analyzer. Degassing of hydrogels was performed at 120 °C for 60 min and then the area analysis was performed using a combination of N_2_ and He gas. N_2_ adsorption isotherms were obtained at −196 °C. The surface area was calculated on the basis of N_2_ gas adsorbed on the surface of hydrogel using multi-point BET method in the relative pressure range of 0.00 to 0.35 [41]. The isotherms were classified according to the International Union of Pure and Applied Chemistry (IUPAC).

#### 4.3.4. Swelling Behavior of Hydrogels

Swelling tests were performed on all formulations of hydrogels by submerging in distilled water until swelling equilibrium was obtained. The hydrogel samples were kept at 37 °C for 24 h, and then withdrawn from water. The excess water from the surface was removed with a paper towel. The weight of swollen hydrogels was measured. The equilibrium swelling ratio was calculated using the Equation (1) [49].

Swelling ratio (g/g) = (W_s_ − W_d_)/W_d_ (1)

where, W_s_ is the weight of the swollen sample and W_d_ is the weight of the dry sample.

#### 4.3.5. In-Vitro Degradation Studies

A fixed area of hydrogels, i.e., 0.1 cm × 0.1 cm was used for in vitro degradation studies. The hydrogel samples of CS/CHI and CS/CHI/Gar were initially weighed and then incubated in phosphate buffer saline (PBS) at 37 °C for 21 days in an orbital shaking incubator (Biobase). Samples were then removed from PBS at the designated time points and then weighed. The weight loss ratio was measured by using Equation (2) [49]. The pH of PBS was measured after 21 days to observe the effect of degradation of different hydrogels on the pH of PBS.

Weight loss (%) = (W_i_ − W_f_)/W_i_ × 100 (2)

#### 4.3.6. Antibacterial Analysis

The disc diffusion method was used to analyze the antibacterial effect of the hydrogels against two bacterial strains; *Escherichia coli* (*E. coli*) and *Staphylococcus aureus* (*S. aureus*). Bacterial strains were cultured in nutrient broth for 24 h at 37 °C and OD was set at 0.015 at 600 nm. For antibacterial analysis, 20 µL of one-day old culture of bacteria was spread on nutrient agar plates and garlic-loaded hydrogels were then placed on agar plates followed by 24 hours’ incubation at 37 °C. A zone of inhibition was formed around samples after incubation which indicates the antimicrobial efficacy of samples. The control sample was garlic free hydrogel of same composition. The disc diffusion was conducted in triplicate for all set of samples and the mean values along with the standard deviation were reported.

#### 4.3.7. Cell-Culture Studies

The fibroblasts (NIH3T3-E1) cells were seeded in Dulbecco’s Modified Eagle Medium (DMEM) (Sigma-Aldrich, St Louis, MO, USA) containing 10% heat-inactivated fetal bovine serum (FBS) and 1% penicillin-streptomycin antibiotics (Sigma-Aldrich, St Louis, MO, USA) in a humidified incubator at 37 °C and 5% CO_2_. 

Before starting the experiment, all the CS/CHI hydrogels with and without Gar were sterilized with gamma irradiation doze of 2.5 K-Grey. The samples with equal size of 1 × 1 cm^2^ were cut and placed in a 24-well tissue culture plate. Cells with a density of 5 × 10^4^ cells/well were seeded on control (without hydrogels) and sample groups pure CS, 66CS33CHI, 80CS20CHI, 67CS33CHIGar, and 80CS20CHIGar. After 5 h of seeding, 10 µL of the culture sample were taken from each well and measured using hemocytometer to determine the unattached versus attached cells. The experiment was performed in triplicate.

### 4.4. Statistical Analysis

Statistical analysis was carried out using one-way Analysis of Variance (ANOVA) in Origin pro 8.5™ software, keeping a significance level of *p* < 0.05. Statistical analysis was done for swelling behavior, in vitro degradation and antibacterial analysis of hydrogels. Results were presented as mean values along with the standard deviation.

## Figures and Tables

**Table 1:** Swelling ratio of synthesized hydrogels.

Sample	Average Wet Weight (g)	Average Dry Weight (g)	Difference (g)	Swelling Ratio (%)
67CS33CHI	9.3865	0.3425	9.044	2641
80CS20CHI	12.572	0.453	12.119	2675

**Table 2:** Change in the weight of the hydrogels after 21 days in PBS.

Sample	Initial Weight (g)	Weight at Day 21 (g)	Change in Weight (%)
67CS33CHI	0.026	0.007	73
80CS20CHI	0.028	0.012	57
67CS33CHIGar	0.012	0.001	91
80CS20CHIGar	0.016	0.013	18

**Table 3:** Composition of CS, CHI, Gar and glycerol in hydrogels.

CS	CHI	Glycerol Solution	Gar	Sample Code
20 mL (66.66%) 0.4 gm/s	10 mL (33.34%) 0.4 gm/s	2.5 mL 0.0375 gm/s	0	67CS33CHI
24 mL (80%) 0.48 gm/s	6 mL (20%) 0.24 gm/s	2.5 mL 0.0375 gm/s	0	80CS20CHI
20 mL (66.66%) 0.4 gm/s	10 mL (33.34%) 0.4 gm/s	2.5 mL 0.0375 gm/s	5 mL	67CS33CHIGar
24 mL (80%) 0.48 gm/s	6 mL (20%) 0.24 gm/s	2.5 mL 0.0375 gm/s	5 mL	80CS20CHIGar
